# Exercise-induced changes in hemostasis markers in marathon runners: effects of enzyme supplementation and determinants

**DOI:** 10.3389/fphys.2026.1843994

**Published:** 2026-06-12

**Authors:** Julia Schoenfeld, Viola Grabs, David C. Nieman, Stefan Holdenrieder, Bernhard Haller, Martin Halle, Johannes Scherr

**Affiliations:** 1 Department of Preventive Sports Medicine and Sports Cardiology, TUM School of Medicine and Health, Technical University of Munich, Munich, Germany; 2 DZHK (German Centre for Cardiovascular Research), partner site Munich Heart Alliance, Munich, Germany; 3 Human Performance Laboratory, Appalachian State University, Kannapolis, NC, United States; 4 Institute of Laboratory Medicine, German Heart Centre Munich, Technical University Munich, Munich, Germany; 5 Insitute of AI and Informatics in Medicine, TUM School of Medicine and Health, Technical University of Munich, Munich, Germany; 6 University Center for Prevention and Sports Medicine, Balgrist University Hospital, University of Zurich, Zurich, Switzerland

**Keywords:** cardiovascular adaptation, exercise, fibrinolysis, inflammation, platelet function, thrombosis

## Abstract

**Background:**

Increases in hemostatic markers, indicators of thrombotic risk, have been reported following strenuous exercise; however, determinants of these responses remain poorly understood. This study aimed to determine whether supplementation with hydrolytic enzymes and flavonoids (Wobenzym^®^ [WOB]) modulates exercise-induced hemostatic responses in marathon runners and to identify factors associated with these changes.

**Methods:**

This predefined subanalysis of the Enzy-MagIC trial was conducted within a prospective, randomized, double-blind, placebo-controlled trial. Participants were randomized (1:1) to receive WOB (rutoside 600–1200 mg/day, bromelain 540–1080 mg/day, trypsin 288–576 mg/day) or placebo for 1 week before and 2 weeks post-race, following a predefined dosing regimen. Blood samples were collected at baseline, immediately, 24 h, and 72 h post-race for analysis of D-dimer, plasminogen activator inhibitor-1 (PAI-1), tissue plasminogen activator (tPA), prothrombin fragment 1 + 2 (F1+2), thrombin generation (calibrated automated thrombography; CAT: lag time, peak thrombin, endogenous thrombin potential [ETP]), maximal platelet aggregation induced by adenosine diphosphate (MPADP), and platelet count.

**Results:**

Among 118 male runners (age 42 ± 11 years; WOB n = 64, placebo n = 54), no differences were observed between groups for any hemostatic marker (all p>0.05). D-dimer, PAI-1, tPA, F1+2, MPADP, and platelet count increased immediately post-race (all p<0.001) and returned toward baseline within 24 h. CAT lag time and peak thrombin increased at 24 h (both p<0.05), whereas ETP decreased immediately post-race (p<0.001). In multivariable analyses, ΔtPA was associated with finishing time and age, and ΔCAT lag time with body fat percentage (all p<0.05).

**Conclusion:**

WOB supplementation did not affect post-race hemostasis; however, marathon running induced hemostatic activation, partly driven by clinical and performance-related characteristics.

## 1 Introduction

Elevated markers of hemostatic activation, including coagulation activation, fibrinolytic activity, and platelet activation, are associated with an increased thrombotic risk and cardiovascular events (Smith et al., 2005; Morange et al., 2006; Kannan et al., 2019). While regular moderate physical activity is associated with substantial cardiovascular benefits (Lee et al., 2014) and a reduced risk of thromboembolic diseases (Kunutsor et al., 2020), prolonged and strenuous exercise transiently increases the risk of cardiovascular events, including coronary events, sudden cardiac death, and myocardial infarction in susceptible individuals (Giri et al., 1999; Albert et al., 2000). This is of particular clinical concern given the growing participation of recreational athletes in mass endurance events such as marathons (Reusser et al., 2021). Potentiation of inflammatory pathways and activation of the coagulation cascade have been proposed as potential mechanisms underlying these risks (Franklin et al., 2020). Marathon running elicits a pronounced acute inflammatory response, characterized by elevated cytokines and acute-phase proteins (Scherr et al., 2011). Experimental and clinical evidence has demonstrated a close bidirectional interaction between inflammation and hemostatic activation (Levi and van der Poll, 2010). Inflammatory signaling promotes coagulation through tissue factor expression, suppression of endogenous anticoagulant pathways, and inhibition of fibrinolysis, while activated coagulation pathways may further amplify inflammatory responses (Levi et al., 2003; Levi and van der Poll, 2010). Elevated fibrinogen concentrations as part of the acute-phase response additionally increase plasma viscosity and erythrocyte aggregation, thereby potentially contributing to a transient prothrombotic state following prolonged strenuous exercise (Baskurt and Meiselman, 2024). In line with this inflammatory–hemostatic interplay, prolonged strenuous exercise induces a coordinated yet temporally heterogeneous hemostatic response (Siegel et al., 2001; Hilberg et al., 2002). Although concomitant activation of coagulation and fibrinolysis may initially preserve equilibrium (Sumann et al., 2007), delayed resolution of procoagulant pathways during recovery (Smith, 2003; Ribeiro et al., 2007) may transiently shift the hemostatic balance toward a prothrombotic state in predisposed individuals. Identifying which athletes are at greatest risk of an excessive hemostatic response, and whether this response can be attenuated, is therefore of clinical relevance and may help to improve the understanding of potential risk profiles in this setting.

However, relatively little is known about the factors associated with and predictive of the magnitude of exercise-induced hemostatic responses following strenuous and prolonged exercise. Nutritional supplements containing anti-inflammatory compounds such as bioactive flavonoids and proteolytic enzymes (e.g., bromelain and trypsin) are commonly used in sports settings with the aim of supporting recovery (O’Connor et al., 2022) (e.g., by reducing inflammation and swelling, alleviating pain, and promoting tissue regeneration) and modulating exercise-induced physiological stress. These compounds have also been proposed to modulate hemostatic activity (Maurer, 2001; Olas, 2022; Tamer et al., 2022), yet clinical evidence remains limited. Furthermore, small-scale studies have suggested that subject characteristics (Stratton et al., 1991; van den Burg et al., 1994; van den Burg et al., 2000), performance-related factors (Szymanski and Pate, 1994; Weiss et al., 1998) and inflammation responses (Levi et al., 2004; Verhamme and Hoylaerts, 2009) may influence the extent of exercise-induced hemostatic activation; however, these findings are constrained by limited sample sizes and, in part, by investigations conducted in heterogeneous or general populations, and it remains unclear which factors determine the magnitude of these responses and whether they can be modulated. To date, no adequately powered prospective study has simultaneously examined the effect of nutritional supplementation on exercise-induced hemostatic activation and identified clinical, inflammatory and performance-related predictors of these responses within a well-characterized cohort of endurance athletes.

Therefore, the aim of this predefined subanalysis was twofold: first, to determine whether supplementation with hydrolytic enzymes and flavonoids (Wobenzym^®^ [WOB]) before and after a marathon affects exercise-induced post-race hemostatic responses in a cohort of marathon runners; and second, to identify clinical and performance-related determinants of these hemostatic responses.

## 2 Method and design

### 2.1 Study design

This was a predefined subanalysis of the Enzy-MagIC study (Enzymes, Marathon, runninG, Inflammation, coagulation; NCT01916408), a prospective, randomized, double-blind, placebo-controlled single-center study. A detailed description of the study design has been previously published (Grabs et al., 2014). The primary aim of the study was to determine whether the intake of oral hydrolytic enzymes and flavonoids before and after the marathon attenuates muscle damage and inflammation, counteracts pro-thrombotic changes in hemostasis, and reduces the incidence of upper respiratory tract infections.

The medical ethics committee of the Technical University of Munich, Munich, Germany (reference number 5820/13), and the Bundesinstitut für Arzneimittel und Medizinprodukte (BfArM), Bonn, Germany (number 4039219) approved the study, and all participants provided written informed consent. The study was conducted in accordance with the Declaration of Helsinki and registered at ClinicalTrials.gov (NCT01916408).

### 2.2 Study participants

Men between 20 and 65 years were eligible if they had successfully completed at least one half-marathon in the past and intended to participate in the Munich Marathon in 2013. Exclusion criteria included known cardiac, musculoskeletal, or psychiatric diseases; acute or chronic hepatic, renal, or inflammatory disease; neoplastic conditions; acute or chronic infections; known coagulopathy; lactose intolerance; and known allergy to any component of the study medication or to pineapple, papaya, or kiwi; pharmacologic treatment for diabetes mellitus or hypertension; use of medications or supplements known to affect immune function; and participation in other interventional trials.

### 2.3 Randomization

A pre-generated randomization list with variable block sizes was used to assign participants in a 1:1 ratio to WOB or placebo, stratified by age. Study medication was pre-packaged and coded to ensure allocation concealment.

### 2.4 Intervention

Participants assigned to the WOB group initiated supplementation 1 week prior to the marathon, following a fixed dosing regimen of 3x4 tablets/day until race day, followed by 3x2 tablets/day for 2 weeks post-race. Each tablet contained 90 mg bromelain, 48 mg trypsin, and 100 mg rutoside, corresponding to a daily intake of 540–1080 mg bromelain, 288–576 mg trypsin, and 600–1200 mg rutoside depending on the dosing phase. No dose adjustment based on body weight was applied. Participants allocated to the placebo group received visually identical inert tablets consisting of lactose monohydrate with no active ingredients and followed the same dosing schedule. Adherence was monitored using treatment diaries and tablet counts. Details of the intervention have been published previously (Grabs et al., 2014; Grabs et al., 2017).

### 2.5 Clinical assessments

All participants were assessed at baseline (5 weeks prior to the marathon) after randomization with the following examinations: medical history, physical examination, anthropometry, resting electrocardiography, carotid artery ultrasound, transthoracic two-dimensional echocardiography, laboratory analyses, and cardiopulmonary exercise testing, as described in detail previously (Grabs et al., 2014; Grabs et al., 2017). Laboratory analyses were additionally performed immediately, 24 h, and 72 h post-race, to capture early post-exercise changes. Staff members conducting the evaluations were blinded to group allocation.

Participants were instructed to refrain from fatty food and from polyphenol-rich foods. Dietary intake was documented using a 3-day food record prior to the marathon race. To prevent hyponatremia and maintain carbohydrate availability, participants consumed two sodium-rich gels per hour during the race (PowerBar Gel Extra-Natrium, 0.5 g Na^+^/100 g), providing approximately 54 g of carbohydrates per hour, which were taken together with fluids throughout the race.

Blood samples were drawn from an antecubital vein in a fasting state, except for the sample collected immediately post-race. D-dimer and prothrombin fragment 1 + 2 (F1+2) were measured using commercially available immunoassays (Siemens Healthcare Diagnostics, Germany). Plasminogen activator inhibitor-1 (PAI-1) antigen and tissue plasminogen activator (tPA) antigen were determined by sandwich ELISA (Hyphen BioMed, France). Platelet aggregation was assessed by impedance aggregometry using the ADPtest on a Multiplate^®^ analyzer (Roche Diagnostics, Germany). Thrombin generation was assessed using the calibrated automated thrombography (CAT^®^; Thrombinoscope BV, Maastricht, the Netherlands), with lag time, endogenous thrombin potential (ETP), and peak thrombin concentration analyzed as outcome parameters. Detailed assay characteristics are provided in the **Supplementary Methods**.

### 2.6 Data analysis

Analyses were based on a complete-case subset of the modified intention-to-treat (mITT) population, defined in accordance with the original Enzy-MagIC trial (Grabs et al., 2017) as all participants who completed at least 30 km of the marathon and had valid biomarker assessments at baseline and immediately post-race. For the present analyses, only complete datasets across all four time points were included. Participants with missing values at any time point were excluded, and no imputation procedures were applied.

All parameters were visually inspected for normality, assessed for kurtosis and skewness, and tested using Shapiro-Wilk normality tests. Variables were considered non-normally distributed if indicated by statistical testing and confirmed by visual inspection. Continuous variables are reported as mean ± standard deviation (SD) when normally distributed or as median (interquartile range [IQR]) when not normally distributed. Categorical variables are presented as absolute and relative frequencies. Group differences between faster and slower runners were analyzed using independent samples t-tests or Mann–Whitney U tests, as appropriate. Categorical variables were compared using Fisher’s exact tests.

To evaluate the effect of WOB supplementation on post-marathon hemostatic responses, repeated-measures ANOVA models were performed for each biomarker with fixed effects of group (WOB vs. placebo) as the between-subject factor and time (pre-race, immediately, 24 h and 72 h post-race) as the within-subject factor, including the interaction (group x time). Sphericity was assessed using Mauchly’s test; when violated, Greenhouse–Geisser–corrected degrees of freedom and p-values were reported.

No significant differences were observed between the WOB and placebo groups, and the following analyses were conducted in the combined cohort. Changes over time were further quantified as the difference from baseline to post-race values (Δ, calculated as post-race minus pre-race) to capture individual responses. These changes were analyzed using paired comparisons between baseline and post-race time points, applying Wilcoxon signed-rank tests. Differences between pre- and post-race values were compared between faster and slower runners using Mann–Whitney U tests. Spearman correlation coefficients were used to assess associations between hemostatic markers and clinical characteristics (age, body mass index [BMI], body fat percentage), performance characteristics (peak oxygen uptake [VO₂peak], marathon finishing time), and markers of myocardial injury (high-sensitivity cardiac troponin T [hs-cTnT]) and inflammation (interleukin-6 [IL-6]). Multivariable linear regression models were fitted to the data to identify predictors of exercise-induced changes in hemostatic markers, including age, BMI, body fat percentage, systolic blood pressure, VO_2_peak, and marathon finishing time as covariates. Collinearity was assessed using variance inflation factors (VIF) and model assumptions were checked accordingly.

All statistical analyses were performed using SPSS Statistics for Windows version 25 (IBM Corp., Armonk, NY, United States) and R Statistical Software (version 4.4.0; Foundation for Statistical Computing, Vienna, Austria). A two-sided significance level of 5% was considered for this analysis.

## 3 Results

### 3.1 Participant characteristics

The mITT population consisted of 138 male participants (Grabs et al., 2017). Of these, 20 were excluded due to invalid or missing blood samples, resulting in a final analytic cohort of N = 118 runners (mean age, 42 ± 11 years). This cohort included n = 64 participants assigned to the WOB group and n = 54 to the placebo group. Mean marathon finishing time was 3:45 ± 0:37 h, with an average exercise intensity during the race of 87 ± 6% of maximal heart rate. Participants had completed a median of 4 previous marathons (range 0-45) and had a mean VO₂peak of 51.1 ± 7.6 mL.kg^-1.^min^-1^ (**Table 1**). Baseline characteristics stratified by faster and slower runners are presented in **Supplementary Table 1**.

**Table 1:** Baseline characteristics of the 118 marathon runners stratified by WOB and placebo groups.

Characteristics	Total Cohort	WOB n=64	Placebo n=54
Age, years	42 ± 11	44 ± 11	41 ± 11
Height, cm	179.4 ± 6.8	180.1 ± 6.5	178.6 ± 7.1
Weight, kg	75.3 ± 9.3	75.5 ± 8.9	75.2 ± 9.9
BMI, kg/m^2^	23.4 ± 2.2	23.2 ± 1.9	23.5 ± 2.5
Body fat, %	13.6 ± 4.6	13.7 ± 4.2	13.4 ± 5.0
Systolic blood pressure, mmHg	123.5 ± 11.5	122.8 ± 11.2	124.4 ± 11.9
VO₂peak, mL·kg^-^¹·min^-^¹	51.1 ± 7.6	50.3 ± 7.1	52.1 ± 8.2
Previous marathons, median (min-max)	4 (0–45)	4 (0–25)	6 (0–45)
Training kilometers during the last 10 weeks (km·wk^-1^)	55 ± 22	52 ± 22	58 ± 21
Marathon time (h:mm)	3:45 ± 0:37	3:50 ± 0:34	3:40 ± 0:40
Family history of CVD, n (%)	16 (13.6)	10 (15.6)	6 (11.1)
Smoking, n (%)			
Never	113 (95.8)	61 (95.3)	52 (96.3)
Former	2 (1.7)	1 (1.6)	1 (1.9)
Current	3 (2.5)	2 (3.1)	1 (1.9)

Data are presented as mean ± SD, median (min-max), or n (%).

BMI, body mass index; CVD, cardiovascular disease; VO₂peak, peak oxygen consumption; WOB, Wobenzym.

### 3.2 Effect of WOB supplementation

**Figure 1:**
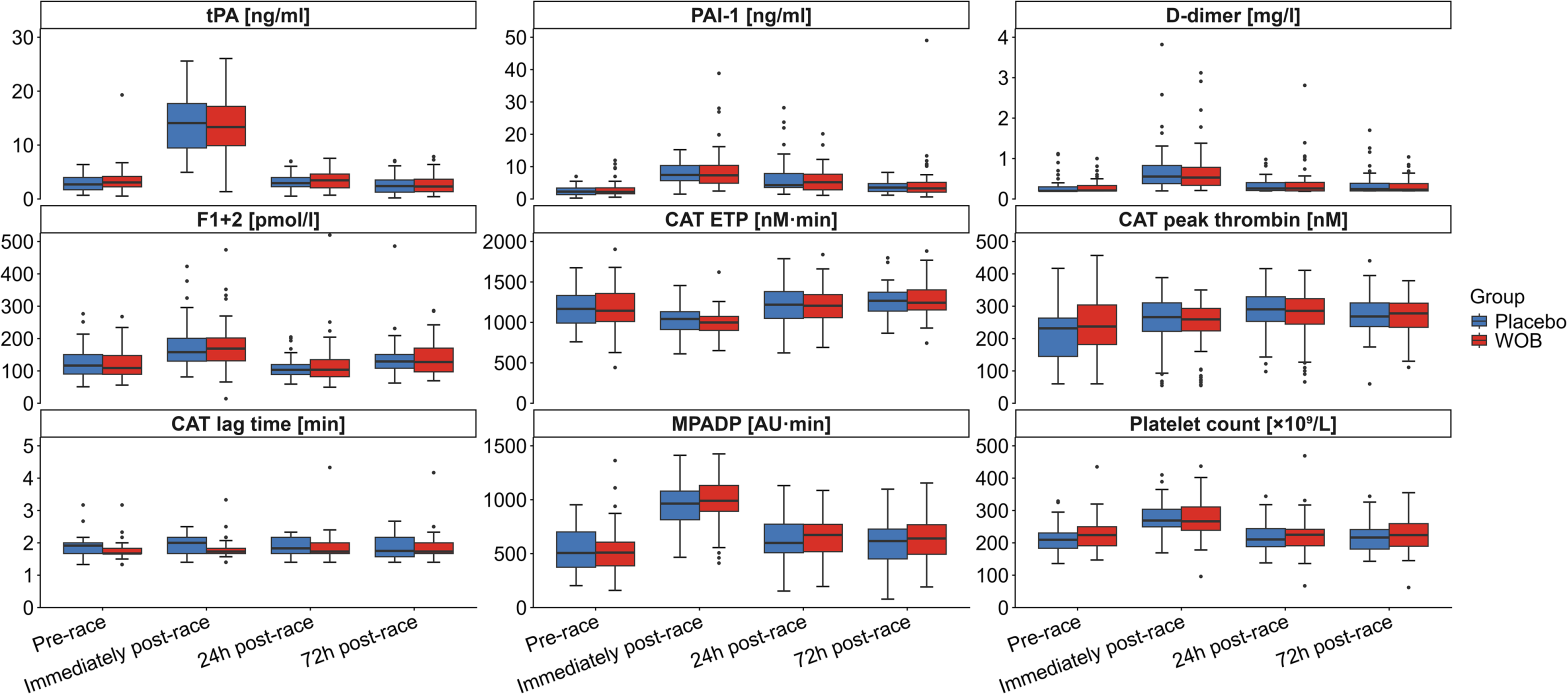
Exercise-induced changes in hemostatic markers stratified by WOB and placebo groups over time. PAI-1, plasminogen activator inhibitor–1; tPA, tissue plasminogen activator; F1+2, prothrombin fragment 1+2; CAT, calibrated automated thrombography; ETP, endogenous thrombin potential; MPADP, maximal platelet aggregation induced by adenosine diphosphate.

No significant group x time interactions were observed for D-dimer, PAI-1, tPA, F1+2, MPADP, platelet count, CAT lag time, CAT peak thrombin, or CAT ETP between the WOB and placebo groups (**Figure 1**, **Table 2**).

**Table 2:** Repeated-measures ANOVA of hemostatic biomarkers before and after marathon running.

Biomarker	Group effect (p)	Time effect (p)	Group × Time (p)
Coagulation activation/thrombin generation			
F1+2	F(1,116) = 0.201, p = 0.655, η²g = 0.0009	F(2.43,281.76) = 38.07, **p < 0.001**, η²g = 0.136	F(2.43,281.76) = 0.353, p = 0.744, η²g = 0.001
CAT lag time	F(1, 74) = 0.56, p = 0.458, η²g = 0.005	F(2.53, 187.56) = 1.10, p = 0.344, η²g = 0.005	F(2.53, 187.56) = 0.77, p = 0.493, η²g = 0.004
CAT peak thrombin	F(1, 113) = 0.06, p = 0.806, η²g < 0.001	F(2.78, 314.4) = 12.51, **p < 0.001**, η²g = 0.063	F(2.78, 314.4) = 2.54, p = 0.061, η²g = 0.013
CAT ETP	F(1, 74) = 0.00, p = 0.996, η²g < 0.001	F(2.41, 178.56) = 27.88, **p < 0.001**, η²g = 0.150	F(2.41, 178.56) = 0.91, p = 0.421, η²g = 0.006
Fibrinolytic system			
D-dimer	F(1, 116) = 0.01, p = 0.914, η²g < 0.001	F(1.84, 213.81) = 36.02, **p < 0.001**, η²g = 0.152	F(1.84, 213.81) = 0.68, p = 0.497, η²g = 0.003
PAI-1	F(1, 116) = 0.58, p = 0.450, η²g = 0.003	F(2.79, 323.94) = 59.63, **p < 0.001**, η²g = 0.195	F(2.79, 323.94) = 2.03, p = 0.115, η²g = 0.008
tPA	F(1, 116) = 0.25, p = 0.617, η²g = 0.001	F(1.34, 155.9) = 505.77, **p < 0.001**, η²g = 0.715	F(1.34, 155.9) = 0.36, p = 0.611, η²g = 0.002
Platelet activation/platelet-related markers			
MPADP	F(1, 116) = 0.46, p = 0.499, η²g = 0.002	F(3, 348) = 176.74, **p < 0.001**, η²g = 0.405	F(3, 348) = 0.45, p = 0.718, η²g = 0.002
Platelet count	F(1, 114) = 1.19, p = 0.278, η²g = 0.009	F(1.67, 190.56) = 152.39, **p < 0.001**, η²g = 0.129	F(1.67, 190.56) = 0.60, p = 0.521, η²g < 0.001

PAI-1, plasminogen activator inhibitor–1; tPA, tissue plasminogen activator; F1 + 2, prothrombin fragment 1+2; CAT, calibrated automated thrombography; ETP, endogenous thrombin potential; MPADP, maximal platelet aggregation induced by adenosine diphosphate. Values represent F statistics with degrees of freedom, p-values, and generalized eta squared (η²g) derived from repeated-measures ANOVA. Values in bold indicate statistically significant effects.

### 3.3 Exercise-induced changes in hemostatic markers

D-dimer, PAI-1, tPA, F1+2, MPADP, and platelet count concentrations increased in nearly all runners from pre-race to immediately post-race (94.9%, 94.1%, 100%, 85.6%, 95.8%, and 96.5% of participants, respectively), with median Δ (interquartile range [IQR]) changes of 0.3 [0.1 to 0.4] mg/L, 5.2 [3.1 to 7.6] ng/mL, 10.3 [6.6 to 14.2] ng/mL, 41.9 [10.9 to 82.4] pmol/L, 430.5 [308.8 to 574.8] AU·min, and 58.0 [34.8 to 82.0] ×10^9^/L, respectively (all p<0.001; **Supplementary Table 2**). Concentrations of D-dimer, PAI-1, tPA, F1+2, MPADP, and platelet count returned near baseline levels 24h post-race (**Supplementary Table 2**). CAT lag time increased slightly from baseline to 24 h post-race (median Δ: 0.1 [-0.1 to 0.2] min; p=0.017). CAT peak thrombin increased immediately post-race (median Δ: 18.0 [-46.9 to 76.6] nM) and peaked at 24 h, whereas CAT ETP decreased immediately post-race (median Δ: -121.4 [-359.0 to 30.8] nM; p<0.001), returned to baseline at 24 h, and exceeded baseline at 72 h (**Supplementary Table 2**). Faster runners showed significantly greater increases from baseline to immediately post-race in Δ tPA (11.9 [8.0 to 16.3] ng/ml vs. 9.1 [5.4 to 12.0] ng/ml**;** p<0.001) compared with slower runners (**Supplementary Table 3**). In addition, applying an age-adjusted D-dimer threshold (age x 10ug/L for participants aged >50 years, standard cut-off of ≥0.5 mg/L for younger participants), 16 of 118 participants (13.6%) had elevated concentrations at baseline, 62 (52.5%) immediately post-race, and 15 (12.7%) and 17 (14.4%) at 24 h and 72 h post-race, respectively. Five participants (4.2%) exhibited persistently elevated D-dimer levels across all time points.

### 3.4 Associations between exercise-induced changes in hemostatic markers and clinical, performance, myocardial injury, and inflammatory markers

**Figure 2:**
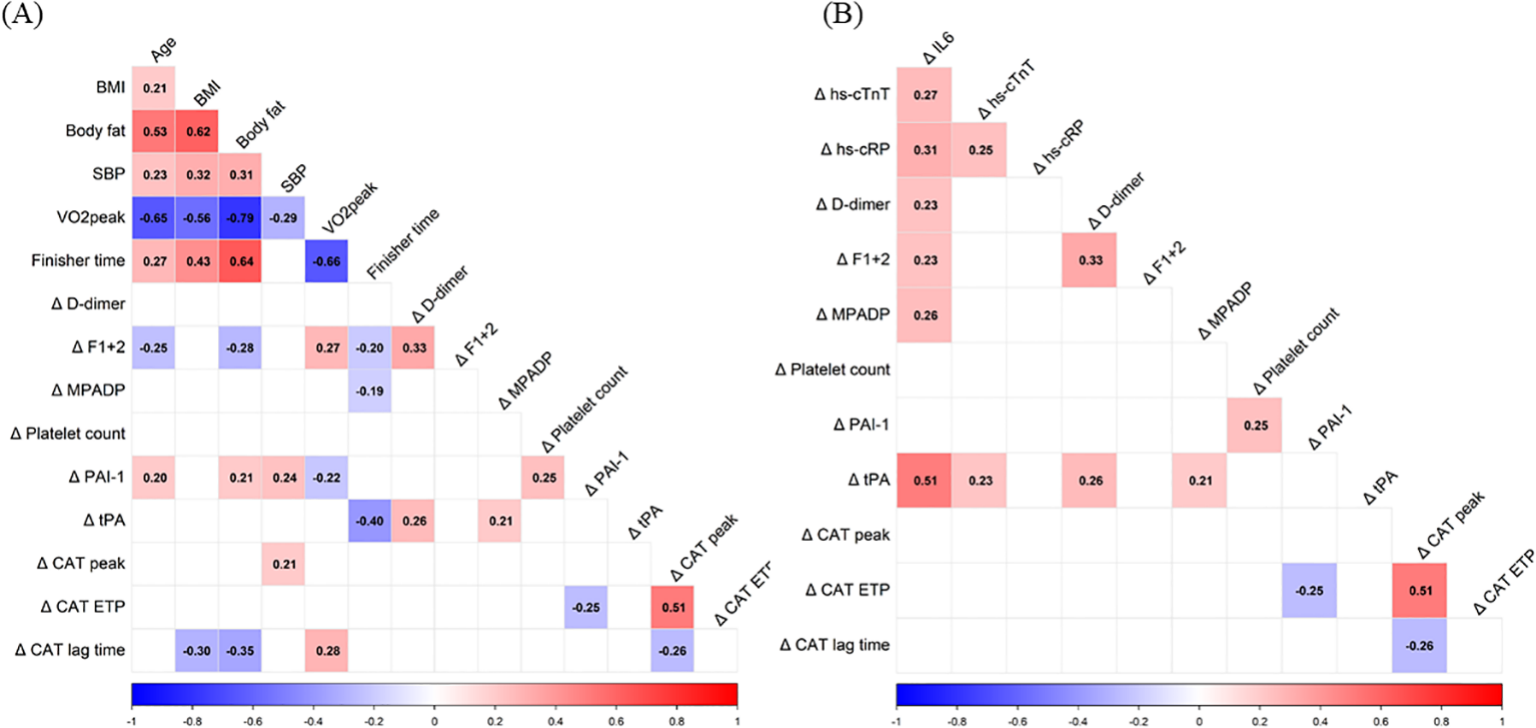
Correlation matrix of changes in hemostatic markers with **(A)** clinical, performance characteristics and **(B)** changes of myocardial damage and inflammation markers. Colors indicate the strength and direction of Spearman correlation coefficients (blue = negative, red = positive). Only statistically significant correlations (p < 0.05) are displayed. Δ values represent changes from baseline to immediately post-race (D-dimer, F1+2, MPADP, PAI-1, tPA, IL-6, hs-cTnT) or baseline to 24h post-race (hs-CRP, CAT peak, CAT lagtime). F1+2, prothrombin fragment 1+2; PAI-1, plasminogen activator inhibitor-1; tPA, tissue plasminogen activator; CAT, calibrated automated thrombography; ETP, endogenous thrombin potential; MPADP, maximal platelet aggregation induced by adenosine diphosphate; hs-cTnT, high-sensitivity cardiac troponin T; IL-6, interleukin-6; hs-CRP, high-sensitivity C-reactive protein.

Changes in D-dimer were positively correlated with changes in F1+2 (r_s_ = 0.33, 95% CI [0.16, 0.49], p<0.001). Changes in tPA were associated with changes in IL-6 (r_s_ = 0.51, 95% CI [0.35, 0.64], p<0.001), and marathon finishing time (r_s_ = -0.40, 95% CI [-0.55, -0.23], p<0.001). Changes in CAT lag time (baseline vs. 24 h post-race) were inversely associated with body fat percentage (r_s_ = -0.35, 95% CI [-0.55, -0.12], p=0.002) and BMI (r_s_ = -0.30, 95% CI [-0.51, -0.06], p=0.008). Further details are provided in **Figure 2**.

### 3.5 Predictors of exercise-induced changes in hemostatic markers

In multivariable linear regression (adjusted R² = 0.223, p<0.001), finisher time was the strongest independent predictor of ΔtPA (baseline to immediately post-race) (β = -0.07, 95% CI [-0.10 to -0.04], p<0.001), indicating that faster runners exhibited greater fibrinolytic activation immediately post-race. Age also showed a significant association with ΔtPA (β = 0.14, 95% CI [0.04 to 0.25], p=0.009), whereas BMI, body fat percentage, systolic blood pressure, and VO₂peak were not independently associated with ΔtPA (all p>.05, **Supplementary Table 4**).

Systolic blood pressure was associated with ΔCAT peak height (baseline to 24 h post-race) (β = 2.13, 95% CI [0.50 to 3.75], p=0.011); however, the overall model did not reach statistical significance (adjusted R²=.05; p=0.086; **Supplementary Table 4**).

For ΔCAT lag time from baseline to 24 h post-race, multivariable regression (adjusted R²=0.14; p=0.009) identified body fat percentage as the only independent predictor (β = -0.036, 95% CI [-0.065 to -0.007], p=0.016). No significant associations were observed for age, systolic blood pressure, VO₂peak, or marathon finish time (all p>0.05; **Supplementary Table 4**).

## 4 Discussion

In this study, we examined whether supplementation with hydrolytic enzymes and flavonoids before and after a marathon affects exercise-induced post-race hemostatic responses in a cohort of marathon runners. Potential determinants of these changes were explored using multivariable modeling. WOB combines the flavonoid rutoside with the proteolytic enzymes bromelain and trypsin, compounds which may influence inflammatory and hemostatic pathways (Maurer, 2001; Olas, 2022; Tamer et al., 2022). In particular, bromelain has demonstrated antithrombotic and fibrinolytic properties in experimental models (Metzig et al., 1999; Azarkan et al., 2020), while flavonoids have been associated with anti-inflammatory and recovery-related effects (Nieman et al., 2020; Carey et al., 2021). However, in our cohort supplementation did not affect exercise-induced changes in hemostatic markers compared with placebo. Evidence on the effects of hydrolytic enzymes and flavonoids on exercise-induced hemostatic responses is limited. Most available data are derived from experimental models (e.g., *in vitro* or animal studies) (Metzig et al., 1999; Azarkan et al., 2020) or non-exercise clinical settings (Taussig and Batkin, 1988; Baumueller and Rau, 2018), limiting direct comparisons with our findings. Even in studies reporting attenuation of post-exercise inflammatory responses following flavonoid supplementation, corresponding effects on coagulation or fibrinolytic markers have not been reported (Carey et al., 2021). This suggests that modulation of inflammatory signaling alone may be insufficient to alter the pronounced hemostatic activation induced by extreme endurance exercise (Sumann et al., 2007). One possible explanation is that the physiological stress imposed by marathon running is so substantial that any potential immunomodulatory effects of supplementation on coagulation pathways remain too small to be detected under these conditions. In other words, the primary driver of hemostatic activation is exercise stress, and nutritional interventions such as WOB supplementation lack the strength to operate as effective countermeasures. Consistent with this interpretation, we also did not observe a significant effect of WOB supplementation on inflammatory parameters in this cohort (Grabs et al., 2017).

### 4.1 Coagulation activation/thrombin generation

**Figure 3:**
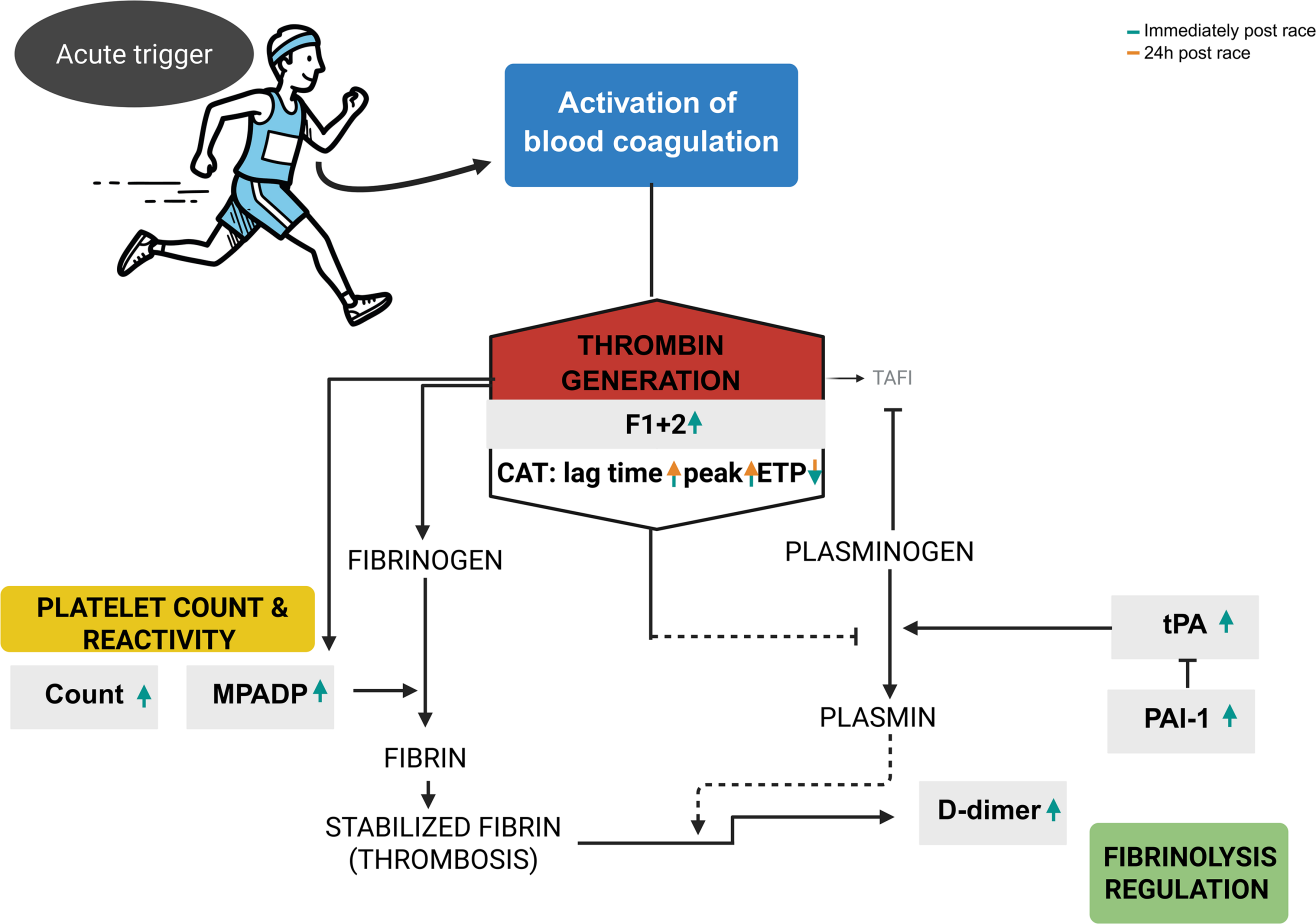
Schematic illustration of marathon-induced changes in hemostasis and related biomarkers. F1+2, prothrombin fragment 1+2; PAI-1, plasminogen activator inhibitor-1; tPA, tissue plasminogen activator; TAFI, thrombin-activatable fibrinolysis inhibitor; CAT, calibrated automated thrombography; ETP, endogenous thrombin potential; MPADP, maximal platelet aggregation induced by adenosine diphosphate.

Independent of supplementation, marathon running resulted in a transient activation of the coagulation system, reflected by increased F1+2 concentrations immediately after the marathon, whereas ex vivo thrombin generation parameters showed a more complex temporal pattern. In our cohort, F1+2 increased post-race and declined toward baseline levels within 24 h (**Figure 3**), consistent with previous reports demonstrating substantial activation of the coagulation system following prolonged endurance exercise. F1+2 is released when prothrombin is cleaved to thrombin, making it a well-established marker of *in vivo* coagulation activation. Previous studies have reported approximately four- to sixfold increases in F1+2 concentrations after marathon running (Prisco et al., 1998; Kupchak et al., 2013). Thrombin generation analysis further showed a prolonged CAT lag time and increased peak thrombin at 24 h post-race, whereas ETP decreased immediately after the marathon (**Figure 3**). Previous studies examining thrombin generation after prolonged endurance exercise have reported heterogeneous results (Hilberg et al., 2002; Cimenti et al., 2013; van der Vorm et al., 2018). Some studies have described reduced ETP immediately after exercise (Hilberg et al., 2002; Cimenti et al., 2013), likely reflecting consumption of coagulation factors or activation of counter-regulatory anticoagulant pathways. In contrast, lower endogenous thrombin potential has also been reported in trained individuals compared with untrained controls (Metzig et al., 1999), suggesting a possible adaptation of the coagulation system to regular endurance training. In this context, the reduction in ETP observed in our cohort may reflect transient consumption of coagulation factors or activation of anticoagulant pathways immediately after the race. In contrast, the increase in peak thrombin at 24 h may reflect recovery of thrombin-generating capacity during early post-exercise recovery. The slightly prolonged lag time observed in our study differs from some reports describing shortened lag times after exercise, but such discrepancies may be related to methodological differences in thrombin generation assays or the timing of blood sampling (van der Vorm et al., 2018).

### 4.2 Fibrinolytic system

In parallel with coagulation activation, markers of fibrinolysis were markedly increased immediately after the marathon. Previous studies have reported two- to threefold increases in D-dimer concentrations immediately after marathon running, with levels remaining elevated for up to 48 h (Prisco et al., 1998; Siegel et al., 2001; Smith et al., 2004; Parker et al., 2012; Kupchak et al., 2013), whereas normalization occurred more rapidly in our cohort. D-dimer elevations after endurance exercise are thought to reflect fibrin formation during coagulation activation and subsequent plasmin-mediated fibrin degradation (Siegel et al., 2001). Similarly, tPA concentrations have been reported to increase three- to fourfold (Prisco et al., 1998; Kupchak et al., 2013) after prolonged endurance exercise, while PAI-1 typically increases two- to fourfold (Prisco et al., 1998; Kupchak et al., 2013). These changes likely reflect exercise-induced epinephrine stimulation of endothelial cells, and endothelial activation and increased shear stress, which promotes the release of fibrinolytic factors from the vascular endothelium.

### 4.3 Platelet activation/platelet-related markers

In addition to changes in coagulation and fibrinolysis, marathon running was associated with increased platelet count and platelet reactivity, reflected by higher MPADP values immediately post-marathon. Exercise-induced platelet activation has been described previously (Röcker et al., 1986; Hanke et al., 2010) and may result from increased catecholamine levels, hemoconcentration, and increased shear stress during strenuous exercise. Inflammatory activation may further contribute to platelet priming and enhanced responsiveness in the post-exercise setting (Skouras et al., 2025). The observed increase in ADP-induced platelet aggregation may reflect both enhanced platelet reactivity and the concomitant increase in circulating platelet count, suggesting that quantitative and qualitative changes in platelets contribute to the overall response. Recent studies have highlighted that acute exercise may transiently increase platelet reactivity, whereas regular training may induce adaptive or modulatory effects on platelet function, indicating a complex and context-dependent regulation of platelet activity in response to physical exercise (Kristiansen et al., 2023).

Together with the activation of coagulation and fibrinolysis, these findings indicate a coordinated but temporally heterogeneous hemostatic response to prolonged endurance exercise, with normalization during recovery. Notably, despite statistically significant changes, F1+2 concentrations remained within the established reference range throughout, whereas D-dimer exceeded the age-adjusted clinical cut-off in 52.5% of participants immediately post-race, suggesting a predominantly physiological rather than pathological hemostatic response in healthy endurance athletes.

### 4.4 Associations

This analysis included n = 118 marathon runners and identified finishing time, age, and body fat as predictors of specific hemostatic responses. Many earlier studies had 10–40 participants, limiting the statistical power to characterize determinants using multivariable modeling. This is an important and relatively understudied area. We observed a positive association between changes in D-dimer and F1+2, suggesting coupled thrombin generation and fibrin turnover. Although previous exercise studies have documented increases in inflammatory markers such as IL-6 (Scherr et al., 2011) and in fibrinolytic markers (tPA), to the best of our knowledge, the association between exercise-induced changes in IL-6 and tPA has not been specifically examined. Our findings therefore provide evidence linking inflammatory activation to the fibrinolytic response in the context of endurance exercise. Changes in CAT lag time were inversely associated with body fat percentage and BMI, with body fat percentage emerging as an independent determinant of thrombin generation dynamics. This observation is consistent with prior evidence demonstrating a procoagulant profile and enhanced thrombin generation in individuals with higher adiposity in non-exercise settings (Chitongo et al., 2017). Previous studies have shown that fibrinolytic activity, including increases in tPA, is more pronounced following high-intensity exercise (Rankinen et al., 1995; Weiss et al., 1998). In line with these findings, marathon finishing time emerged as the strongest determinant of the fibrinolytic response in our study, indicating greater activation in faster runners.

### 4.5 Clinical implications

Although hemostatic alterations following marathon running are generally transient in healthy athletes, prolonged endurance exercise may induce a temporary hypercoagulable state that could become clinically relevant in individuals with additional prothrombotic risk factors (Miele et al., 2024). Case reports and observational studies have reported venous thromboembolism in otherwise healthy marathon runners following endurance events (Hull et al., 2015; Sanz de la Garza et al., 2017), with dehydration, hemoconcentration, endothelial activation, and prolonged post-race immobility proposed as contributing mechanisms (Parker et al., 2012; Taylor et al., 2019). Of particular concern is a potential “second-hit” scenario in which exercise-induced hemostatic activation is compounded by prolonged immobilization during post-race travel, potentially amplifying thrombotic risk (Skouras and Koulouvaris, 2025). The present findings extend previous observations by providing temporal resolution of hemostatic responses up to 72 h post-race and by demonstrating substantial interindividual variability in these responses. This is clinically relevant for biomarker interpretation, as D-dimer concentrations remained elevated for up to 24 h post-race in the present cohort, potentially generating false-positive results when performing VTE evaluation in athletes presenting to emergency departments shortly after endurance events (Tripodi et al., 2026). In addition, faster race performance and older age were associated with greater fibrinolytic activation, whereas higher body fat percentage predicted attenuated thrombin generation responses. These findings suggest that exercise-induced hemostatic responses are modified by individual athlete characteristics, including performance level, age, and cardiometabolic body composition. The association between older age and greater tPA release may reflect compensatory fibrinolytic activation in response to increased baseline coagulability (Gudnason et al., 2003), while higher adiposity may indicate a more chronically altered coagulation profile associated with impaired fibrinolysis and excess PAI-1 activity (La Rosa et al., 2025). Taken together, these findings highlight that recent endurance exercise and individual athlete characteristics should be considered when interpreting hemostatic biomarkers in sports medicine. The observed variability and prolonged recovery kinetics suggest that thrombotic vulnerability following strenuous exercise may differ substantially between athletes. These findings support future studies aimed at individualized thrombotic risk stratification in endurance athletes.

### 4.6 Limitations

This study represents a predefined subanalysis of the Enzy-MaGIC trial (Grabs et al., 2014), which included only male participants to avoid potential confounding by sex-specific hormonal fluctuations known to influence several of the investigated parameters. Consequently, the findings of this analysis cannot be generalized to female runners. Second, the biological activity and systemic availability of the supplemented hydrolytic enzymes and flavonoids were not directly assessed. Previous studies have demonstrated the bioavailability of these compounds in humans, supporting their systemic exposure following oral supplementation. Third, this study focused on biochemical and functional hemostatic markers and did not assess clinical thrombotic outcomes or viscoelastic whole-blood coagulation profiles. Therefore, the clinical implications of the observed exercise-induced hemostatic activation cannot be directly inferred. In addition, established diagnostic cut-offs are available for D-dimer, whereas comparable thresholds are lacking for other hemostatic markers, limiting their direct clinical interpretability. Importantly, these cut-offs were derived from thrombosis-suspicion settings rather than exercise contexts, and age-adjusted thresholds may further influence the proportion of participants classified as elevated. Fourth, analyses were restricted to participants with complete datasets across all time points, and no imputation procedures were applied. While this approach ensured data completeness and internal consistency, it may have limited statistical power. Nevertheless, this study represents one of the largest investigations in this field to date. Fifth, the findings are specific to marathon runners and may not be generalizable to other endurance disciplines, training loads, or less-trained populations. Sixth, fluid intake during the race, hydration status, and hematocrit changes were not systematically assessed. Therefore, potential plasma volume shifts may have influenced biomarker concentrations and hemostatic parameters. Finally, given the number of statistical tests performed, the risk of type I error cannot be excluded, as no formal adjustment for multiple comparisons was applied and results should therefore be interpreted as exploratory.

## 5 Conclusion

In conclusion, ingestion of oral hydrolytic enzymes and flavonoids before and after a marathon did not affect post-race hemostasis. However, marathon running elicited a transient hemostatic response characterized by concomitant procoagulant activation and an increased fibrinolytic response. This produced a transient procoagulant state that appeared to be counterbalanced by simultaneous fibrinolytic activation, maintaining overall hemostatic balance. These alterations largely resolved within 24 h, with part of the inter-individual variability explained by differences in age, finishing time, and body fat percentage. These findings are consistent with the published literature showing that marathon running produces transient activation of coagulation, fibrinolysis, and platelet pathways. This study used a strong research design and a large sample size, and provided meaningful new evidence through the inclusion of thrombin-generation assays and an analysis of the determinants of individual variability.

## Data Availability

The datasets presented in this article are not readily available because of ethical and data protection regulations. Requests to access the datasets should be directed to the corresponding author.

## Supplementary material

Supplementary Table 1 Baseline characteristics of faster and slower runners.

Supplementary Table 2 Hemostatic markers at baseline, immediately post-race, 24 h and 72 h post-race.

Supplementary Table 3 Comparison of hemostatic markers between faster and slower runners at baseline, immediately, 24 h and 72 h post-race.

Supplementary Table 4 Predictors of exercise-induced changes in hemostatic markers.
