# Supplementary material for: Exercise-induced changes in hemostasis markers in marathon runners: effects of enzyme supplementation and determinants
Source: Front Physiol. 2026 Jun 12;17:1843994. doi: 10.3389/fphys.2026.1843994 (PMC13303608; doi:10.3389/fphys.2026.1843994)
Supplement: Supplementary Table 1 — Baseline characteristics of faster and slower runners. [file Supplementaryfile1.docx]

Supplementary Material

# Supplementary Material

Biomarker analysis

D-dimer was measured quantitatively using a particle-enhanced immunoturbidimetric assay (INNOVANCE® D-Dimer, Siemens Healthcare Diagnostics, Germany) on the BCS® XP coagulation analyzer. The measuring range of the assay is 0.17 – 4.40 mg/L FEU, with a limit of detection (LoD) of 0.05 mg/L FEU. The interassay coefficient of variation ranged from 2.2% to 7.9%, depending on concentration. The upper reference limit (URL) in healthy volunteers (90^th^ percentile) is 0.55 mg/L FEU. For clinical interpretation, an age-adjusted D-dimer threshold was applied (≥0.5 mg/L for participants aged ≤50 years; age × 10 µg/L for participants aged >50 years), in accordance with ADJUST-PE criteria^1^. Prothrombin fragment 1+2 (F1+2) was measured using a commercially available sandwich enzyme immunoassay (Enzygnost® F1+2 monoclonal, Siemens Healthcare Diagnostics, Germany). The measuring range of the assay is 20–1200 pmol/L. The interassay coefficient of variation is 8.0%. The reference range in citrated plasma of healthy adults has a median of 115 pmol/L. Plasminogen activator inhibitor-1 (PAI-1) antigen was measured using a commercially available sandwich ELISA (ZYMUTEST® PAI-1 Antigen, Hyphen BioMed, France). The measuring range of the assay is 1–25 ng/mL. The interassay coefficient of variation ranged from 5–10%. Tissue plasminogen activator (tPA) antigen was measured using a commercially available sandwich ELISA (ZYMUTEST® tPA Antigen, Hyphen BioMed, France). The measuring range of the assay is 0–20 ng/mL. The interassay coefficient of variation is 5.0%. Platelet aggregation was assessed by impedance aggregometry using the ADPtest on a Multiplate® analyzer (Roche Diagnostics, Germany). Citrated whole blood was stimulated with adenosine diphosphate (final concentration 6.5 µM), and aggregation was quantified as area under the curve (AUC, AU*min). The reference range (2.5th–97.5th percentile) for citrated blood is 480 – 1190 AU*min. Thrombin generation was assessed using the calibrated automated thrombography (CAT®; Thrombinoscope BV, Maastricht, the Netherlands). Thrombin generation in plasma was measured by a fluorogenic substrate in the presence of a thrombin calibrator.

# Supplementary Figures and Tables

## Supplementary Table

**Supplementary Table 1** Baseline characteristics of faster (marathon finisher time<3:44 h) and slower runners (marathon finisher time ≥3:44 h)

| **Characteristics** | **Faster runners** | **Slower runner** | **P value** |
| --- | --- | --- | --- |
| Age, years | 39 ± 11 | 45 ± 9.7 | **.003** |
| Height, cm | 179.2 ± 7.0 | 179.7 ± 6.8 | .699 |
| Weight, kg | 72.5 ± 8.5 | 78.3 ± 9.3 | **<.001** |
| BMI, kg/m^2^ | 22.5 ± 1.7 | 24.2 ± 2.3 | **<.001** |
| Body fat, % | 10.9 ± 3.7 | 16.2 ± 3.9 | **<.001** |
| Systolic blood pressure, mmHg | 122.2 ± 11.3 | 124.6 ± 11.7 | .263 |
| VO_2_peak, mL·kg⁻¹·min⁻¹ | 55.7 ± 6.8 | 46.7 ± 5.5 | **<.001** |
| Previous marathons, median (min-max) | 6 (0-37) | 3 (0-45) | .094 |
| Training kilometers during the last 10 weeks (km^.^wk^-1^) | 62 ± 24 | 48 ± 18 | **<.001** |
| Marathon time (h:mm) | 3:16 ± 0:20 | 4:13 ± 0:27 | **<.001** |
| Family history of CVD, n (%) | 4 (7.1) | 12 (20.7) | **.044** |
| Smoking, n (%) |  |  | 1.000 |
| Never | 54 (96.4) | 56 (94.9) |  |
| Former | 1 (1.8) | 1 (1.7) |  |
| Current | 1 (1.8) | 2 (3.4) |  |

Data are presented as mean ± SD, median (min-max), or n (%).

Abbreviations: BMI, body mass index; CVD, cardiovascular disease; VO_₂_peak, peak oxygen consumption.

**Supplementary Table 2** Hemostatic markers at baseline, immediately post-race, 24 h and 72 h post-race

|  | **Median [IQR] and Sample size** | | | |  | **p-Value** | | |
| --- | --- | --- | --- | --- | --- | --- | --- | --- |
|  | **Baseline** | **Immediately** | **24 h post-race** | **72 h post-race** |  | Baseline vs. Immediately | Baseline vs.  24 h post | Baseline vs. 72 h post |
| **Coagulation activation / thrombin generation** | | | | | |  |  |  |
| F1+2, pmol/l | 114.7  [90.3-148.9] | 162.8  [130.2-201.4] | 103.6  [83.6-129.0] | 129.3  [100.5-156.1] | 118 | **<.001** | **.002** | **.004** |
| CAT lag time, min | 1.7  [1.7-2.0] | 1.8  [1.7-2.1] | 1.8  [1.7-2.0] | 1.7  [1.7-2.1] | 79 | .105 | **.017** | .271 |
| CAT peak thrombin, nM | 234.6  [173.0-292.9] | 260.1  [217.9-299.0] | 288.1  [246.2-325.1] | 274.6  [237.3-310.5] | 115 | .108 | **<.001** | **<.001** |
| CAT ETP, nM·min | 1160.9  [992.2-1343.7] | 1017.5  [905.8-1097.6] | 1206.0  [1051.2-1347.9] | 1252.2  [1150.3-1385.1] | 79 | **<.001** | .352 | **.002** |
| **Fibrinolytic system** | |  |  |  |  |  |  |  |
| D-Dimer, mg/l | 0.2  [0.2-0.3] | 0.5  [0.3-0.8] | 0.3  [0.2-0.4] | 0.2  [0.2-0.4] | 118 | **<.001** | **.005** | .048 |
| PAI-1, ng/ml | 2.3  [1.5-3.4] | 7.4  [4.9-10.4] | 4.8  [3.4-7.9] | 3.5  [2.2-5.0] | 118 | **<.001** | **<.001** | **<.001** |
| tPA, ng/ml | 2.9  [2.0-4.0] | 13.6  [9.7-17.6] | 3.0  [2.1-4.3] | 2.4  [1.3-3.6] | 118 | **<.001** | .186 | **.002** |
| **Platelet activation / platelet-related markers** | | | | | |  |  |  |
| MPADP, **AU·min** | 507.0  [378.2-675.5] | 977.0  [838.8-1086.8] | 624.0  [508.2-771.8] | 630.0  [477.2-739.8] | 118 | **<.001** | **<.001** | **<.001** |
| Platelet count, ×10⁹/L | 211.5  [183.8–239.5] | 267.5  [240.2–307.2] | 220.5  [188.8–244.0] | 219.0  [189.0–254.2] | 116 | **<.001** | **.040** | **.013** |

Abbreviations: PAI-1, plasminogen activator inhibitor–1; tPA, tissue plasminogen activator; F1+2, prothrombin fragment 1+2; CAT, calibrated automated thrombography; ETP, endogenous thrombin potential; MPADP, maximal platelet aggregation induced by adenosine diphosphate; IQR, interquartile range.

**Supplementary Table 3** Comparison of hemostatic markers between faster (finisher time<3:44 h) and slower runners (finisher time ≥3:44 h) at baseline, immediately, and 24 h and 72 h post-race

|  | **Faster runner** | | | | **Slower runner** | | | | **Faster vs. Slower** |
| --- | --- | --- | --- | --- | --- | --- | --- | --- | --- |
|  | Baseline | Immediately | 24 h | 72 h | Baseline | Immediately | 24 h | 72 h | Baseline vs. Immediately |
| **Coagulation activation / thrombin generation** | | | | | |  |  |  | Δ median [IQR];  p value |
| **F1+2, pmol/l** | 101.2  [87.0-143.6] | 161.1  [130.6-214.2] | 98.4  [76.5-116.9] | 118.0  [95.2-149.8] | 122.0  [95.9-155.3] | 160.1  [125.0-191.9] | 111.1  [90.4-136.3] | 137.9  [110.2-164.9] | 51.1 [20.9-88.6] vs. 30.6 [6.1-74.0];  .082 |
| **CAT lag time, min** | 1.7  [1.7-1.9] | 1.8  [1.7-2.1] | 1.8  [1.7-2.1] | 1.7  [1.5-2.0] | 1.7  [1.7-2.0] | 1.8  [1.7-2.0] | 1.8  [1.7-2.0] | 1.8  [1.7-2.1] | 0.0 [-0.1-0.2] vs. 0.1 [0.1-0.2];  .704 |
| **CAT peak height, nM** | 235.1  [166.4-291.0] | 272.3  [227.5-310.3] | 270.0  [225.7-313.5] | 273.0  [230.6-307.3] | 234.5  [178.4-295.6] | 250.1  [216.4-284.8] | 292.9  [258.7-330.6] | 271.3  [237.9-311.1] | 26.2 [-27.3-78.3] vs. 7.5 [-58.6-71.8];  .343 |
| **CAT ETP, nM·min** | 1112.3  [1000.4-1253.4] | 998.8  [906.8-1072.3] | 1124.5  [1012.5-1244.4] | 1237.1  [1167.4-1330.5] | 1222.9  [991.0-1347.9] | 1037.4  [876.8-1106.4] | 1243.7  [1068.0-1456.8] | 1247.6  [1150.3-1399.4] | -159.2 [-343.6-3.5] vs. -101.3 [-375.6-31.2];  .882 |
| **Fibrinolytic system** | | |  |  |  |  |  |  |  |
| **D-Dimere, mg/l** | 0.2  [0.2-0.3] | 0.5  [0.3-0.9] | 0.3  [0.2-0.4] | 0.2  [0.2-0.4] | 0.2  [0.2-0.4] | 0.5  [0.3-0.7] | 0.3  [0.2-0.4] | 0.3  [0.2-0.4] | 0.3 [0.1-0.5] vs. 0.2 [0.1-0.4];  .561 |
| **PAI-1, ng/ml** | 1.9  [1.2-2.6] | 6.9  [5.0-8.9] | 3.8  [2.8-5.5] | 2.7  [1.8-4.3] | 2.9  [1.9-3.8] | 8.7  [5.1-10.8] | 6.5  [3.9-8.8] | 3.8  [3.0-5.5] | 5.0 [3.5-7.0] vs. 5.5 [2.87-8.4];  .724 |
| **tPA, ng/ml** | 2.5  [1.7-3.5] | 15.0  [10.4-18.8] | 2.6  [1.6-3.8] | 1.6  [1.0-2.7] | 3.3  [2.5-4.8] | 13.3  [9.2-16.1] | 3.7  [2.5-4.7] | 3.1  [1.9-4.2] | 11.9 [8.1-16.3] vs. 9.1 [5.4-11.9];  **<.001** |
| **Platelet activation / platelet-related markers** | | | | |  |  |  |  |  |
| **MPADP, AU·min** | 506.5  [392.8-609.2] | 1005.5  [868.5-1119.5] | 618.0  [508.3-743.0] | 661.5  [485.5-731.5] | 521.0  [371.5-701.0] | 964.0  [790.0-1083.0] | 631.0  [514.0-809.0] | 608.0  [470.0-765.0] | 452.0 [355.5-603.2] vs. 390.0 [265.0-573.5];  .146 |
| **Platelet count,** ×10⁹/L | 206  [184–236] | 267  [242–304] | 216 [186–242] | 217 [184–250] | 226  [180–243] | 271  [242–311] | 224  [191–254] | 220 [192–259] | 61.0 [42.0–82.0] vs. 57.0 [34.0–87.0];  .599 |

Abbreviations: PAI-1, plasminogen activator inhibitor–1; tPA, tissue plasminogen activator; F1+2, prothrombin fragment 1+2; CAT, calibrated automated thrombography; ETP, endogenous thrombin potential; MPADP, maximal platelet aggregation induced by adenosine diphosphate; IQR, interquartile range.

**Supplementary Table 4** Predictors of exercise-induced changes in hemostatic markers

| **Model** | **Predictor** | **Regression coefficient β** | **95% CI** | **P-Value** |
| --- | --- | --- | --- | --- |
| ΔtPA  (R² = .223) | Age | 0.14 | 0.04 to 0.25 | **.009** |
|  | BMI | 0.18 | –0.34 to 0.69 | .502 |
|  | Body fat % | –0.02 | –0.34 to 0.29 | .887 |
|  | Systolic blood pressure | –0.01 | –0.08 to 0.07 | .872 |
|  | VO₂peak | 0.04 | –0.16 to 0.25 | .666 |
|  | Finishing time | –0.07 | –0.10 to –0.04 | **<.001** |
| ΔCAT peak height  (R² = .05) | Age | 1.09 | –1.13 to 3.32 | .332 |
|  | BMI | 2.65 | –8.06 to 13.37 | .625 |
|  | Body fat % | 1.73 | –4.76 to 8.23 | .598 |
|  | Systolic blood pressure | 2.13 | 0.50 to 3.75 | **.011** |
|  | VO₂peak | 3.67 | –0.57 to 7.91 | .089 |
|  | Finishing time | 0.24 | –0.40 to 0.89 | .457 |
| ΔCAT lag time  (R² = .14) | Age | 0.005 | –0.005 to 0.015 | .338 |
|  | BMI | –0.017 | –0.063 to 0.029 | .462 |
|  | Body fat % | –0.036 | –0.065 to –0.007 | **.016** |
|  | Systolic blood pressure | 0.003 | –0.004 to 0.010 | .422 |
|  | VO₂peak | 0.006 | –0.015 to 0.026 | .570 |
|  | Finishing time | 0.002 | –0.001 to 0.005 | .149 |

Abbreviations: BMI, body mass index; tPA, tissue plasminogen activator; CAT, calibrated automated thrombography; VO_₂_peak, peak oxygen consumption; Δ values represent changes from baseline to immediately post-race (tPA) and baseline to 24h post-race (CAT peak, CAT lag time).

**
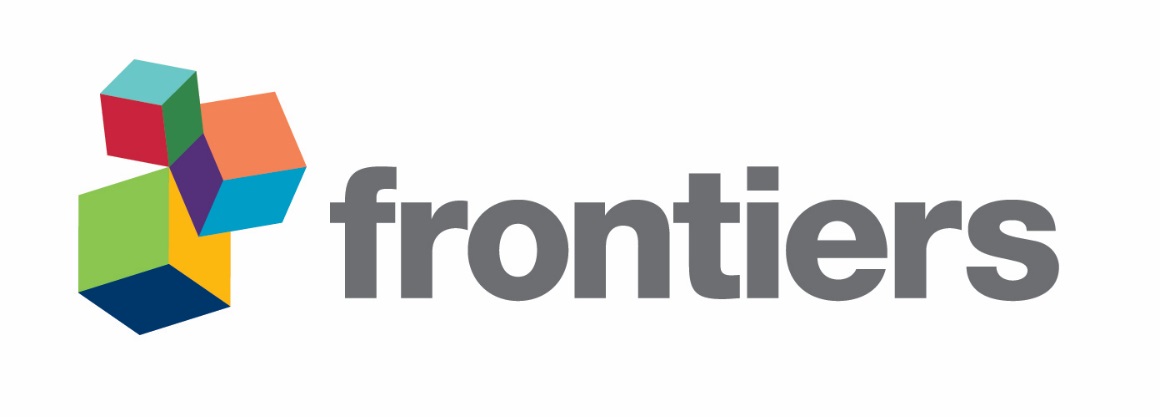
**

References

1. Righini M, Van Es J, Den Exter PL, et al. Age-adjusted D-dimer cutoff levels to rule out pulmonary embolism: the ADJUST-PE study. *Jama*. Mar 19 2014;311(11):1117-24. doi:10.1001/jama.2014.2135
